# Eco‐Friendly Nanoplatforms for Crop Quality Control, Protection, and Nutrition

**DOI:** 10.1002/advs.202004525

**Published:** 2021-03-03

**Authors:** Chao‐Yi Wang, Jie Yang, Jian‐Chun Qin, Ying‐Wei Yang

**Affiliations:** ^1^ College of Chemistry and College of Plant Science Jilin University Changchun 130012 P. R. China

**Keywords:** crop growth regulation, nanomaterials, pest control, pesticides detection, phytopathogen inactivation

## Abstract

Agricultural chemicals have been widely utilized to manage pests, weeds, and plant pathogens for maximizing crop yields. However, the excessive use of these organic substances to compensate their instability in the environment has caused severe environmental consequences, threatened human health, and consumed enormous economic costs. In order to improve the utilization efficiency of these agricultural chemicals, one strategy that attracted researchers is to design novel eco‐friendly nanoplatforms. To date, numerous advanced nanoplatforms with functional components have been applied in the agricultural field, such as silica‐based materials for pesticides delivery, metal/metal oxide nanoparticles for pesticides/mycotoxins detection, and carbon nanoparticles for fertilizers delivery. In this review, the synthesis, applications, and mechanisms of recent eco‐friendly nanoplatforms in the agricultural field, including pesticides and mycotoxins on‐site detection, phytopathogen inactivation, pest control, and crops growth regulation for guaranteeing food security, enhancing the utilization efficiency of agricultural chemicals and increasing crop yields are highlighted. The review also stimulates new thinking for improving the existing agricultural technologies, protecting crops from biotic and abiotic stress, alleviating the global food crisis, and ensuring food security. In addition, the challenges to overcome the constrained applications of functional nanoplatforms in the agricultural field are also discussed.

## Graphical abstract

The recent advance of nanoplatforms with functional components for pesticides and mycotoxins on‐site detection, phytopathogen inactivation, pest control, and crops growth regulation in the agricultural field are summarized, and the prospects and challenges of nano‐based platforms for the sustainable development of green agriculture are also discussed.

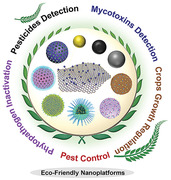

## 1 Introduction

Agricultural chemicals, including herbicides, insecticides, fungicides, as well as fertilizers play an indispensable role in traditional farming methods, which have made significant contributions to increasing crop yields, meeting the food demand of the escalating global population, and ensuring the sustainable development of modern agriculture.^[^ ^1^ ^]^ However, the wide and indiscriminate use of these agricultural chemicals causes an enormous influence on the quality of global agricultural products and ecological environment. More than 90% of the substances ultimately fail to reach target organisms, but are lost by volatilization, binding with minerals/organic substances in the soil or leaching into the water bodies. As a result, these residues of agricultural chemicals remaining in the soil and water lead to the generation of drug resistance in target organisms, destroying the biodiversity of the ecosystems.^[^ ^2^ ^]^ On the other hand, mycotoxins, as the secondary metabolites produced by fungi specie, are a serious pollutant that contaminates 25% of the global food crops, causing the loss of 1 billion metric tons of crop yields annually.^[^ ^3^ ^]^ Moreover, it is well documented that the ingestion of mycotoxins‐infected agricultural commodities poses a severe threat to human health.^[^ ^4^ ^]^

Besides, plant pathogens, including fungi, bacteria, and virus, have caused enormous both pre‐ and post‐harvest losses to the crop yields. Due to the serious impact of plant health problems, nearly 30–40% of global crop yields are lost annually.^[^ ^5^ ^]^ For instance, garden asparagus has been cultivated for more than 2500 years, which is regarded as the king of vegetables as well as an important commercial crop in China. However, *Phomopsis asparagi* (*P. asparagi*), as a kind of fungus, can cause asparagus stem blight, which is a major obstacle to asparagus output on global trade.^[^ ^6^ ^]^ Currently, with continuous spread of stem blight, fungicides have been applied for its management. The use of fungicides seems to be an effective method to control the crops disease and reduce the loss of crops during harvest, but the frequent use of the synthetic fungicides against plant pathogens will break the environmental balance, and human beings have paid more and more attention to plant pathogens resistance and health risks. Besides, as the major contributors to modern agriculture, the advent of chemical insecticides has indeed paved a way to increase crop production and alleviating biotic stress, such as pest invasion.^[^ ^7^ ^]^ However, the inappropriate use of insecticides can increase pest outbreaks and crops losses due to pest resistance and unintentional destruction of natural enemies of pests.^[^ ^8^ ^]^ Eventually, the excessive use of chemical insecticides will result in the emergence of new drug resistance in the course of time thus induce harmful impacts on food safety, human health, and ecosystems.^[^ ^9^ ^]^

Additionally, because of a sessile lifestyle, crops continuously encounter adverse abiotic stresses, including drought,^[^ ^10^ ^]^ salinity,^[^ ^11^ ^]^ extreme temperatures,^[^ ^12^ ^]^ nutrient deficiency,^[^ ^13^ ^]^ and heavy metal pollution,^[^ ^14^ ^]^ which are serious threat to agricultural productivity.^[^ ^15^ ^]^ For instance, under natural conditions, the primary osmotic stress faced by plants, such as drought and/or salinity stresses, may cause physiological changes in plants, including cellular damage, homeostasis disorder, and ion disequilibrium, ultimately affecting the yield and quality of crops. Although crops have evolved a series of characteristics to overcome harsh natural environment, many researches are still in a long and hard process to investigate the resistance genes of the crops. In addition, due to the difficulty of the accurate management, numerous fertilizers have been used to improve the agricultural gains in the face of environmental stress. However, long‐term overfertilization will leave fertilizers in the soil, resulting in severe soil acidification and environmental degradation. Therefore, there is an ongoing and imperative need to provide a sustainable strategy to regulate crops growth when they fail to withstand environmental stress.

**Figure 1:**
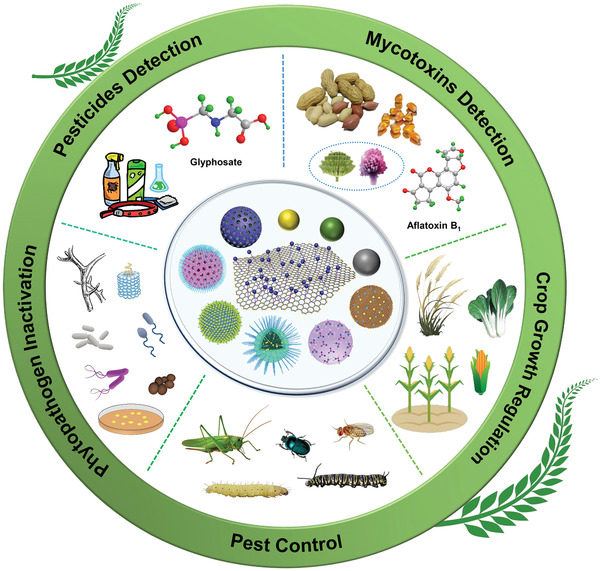
Schematic representation of some advanced nanoplatforms with specific properties for agricultural applications, including on‐site pesticides and mycotoxins detection, phytopathogen inactivation, pest control, and crops growth regulation.

Over the past decades, to conquer the current challenges of modern agriculture, advanced materials have been employed to fabricate eco‐friendly nanoplatforms with unique properties for sustainable agricultural development, such as silica‐based materials, metal/metal oxide nanoparticles (NPs), carbon nanoparticles (CNs), upconversion nanoparticles (UCNPs), and clay mineral, showing bright prospects and enhanced effects in pesticides and mycotoxins on‐site detection, phytopathogen inactivation, pest control, and crop growth regulation (**Figure** **1**).

Among these advanced nano‐carriers for the delivery of agricultural chemicals, mesoporous silica NPs (MSNs) are one of the most commonly and widely used materials for the construction of cargo delivery systems since 2001, due to its high loading capacity, tunable pore size, good biocompatibility, large surface area, and easy functionalization.^[^ ^16^, ^17^ ^]^ Moreover, MSNs can be quickly captured into the leaves of crops, and then transferred to stems, petioles and roots, improving the utilization efficiency of pesticides.^[^ ^18^ ^]^ Additionally, the surfaces of MSNs can be easily functionalized by gated materials to respond to a variety of exogenous and endogenous stimuli, such as pH,^[^ ^16^, ^19^ ^]^ light,^[^ ^20^ ^]^ temperature,^[^ ^21^ ^]^ enzymes,^[^ ^22^, ^23^ ^]^ and competitive agents,^[^ ^24^ ^]^ allowing cargo molecules to be released from the pores of MSNs in a controlled manner.^[^ ^25^ ^]^ Therefore, MSNs‐based smart cargo delivery systems with unique structural characteristics have been designed and applied in agricultural chemicals delivery protocols via adsorption, encapsulation, and/or conjugation of chemical ingredients, which can release fertilizers or pesticides in a slow and continuous manner for longer durations to promote or protect plant growth.^[^ ^7^, ^16^, ^26^ ^]^

In addition, metal/metal oxide NPs have been widely used as functional materials for imaging,^[^ ^27^ ^]^ sensing,^[^ ^28^ ^]^ catalysis,^[^ ^29^ ^]^ antimicrobial,^[^ ^30^ ^]^ virus detection,^[^ ^31^ ^]^ and medicine.^[^ ^32^ ^]^ Notably, the excellent properties of these NPs are significantly originated from their optical property, magnetism, small size, surface plasmons, and low toxicity.^[^ ^33^ ^]^ Recently, metal/metal oxide NPs, as one of the most prevailing materials, have attracted much attention in modern agricultural field, due to their remarkable antimicrobial activity, even at extremely low concentrations.^[^ ^34^ ^]^ Metal/metal oxide NPs based on Ag, Mg, Zn, and Ti are the most widely used fungicides to induce the generation of reactive oxygen species (ROS) against phytopathogen. Besides, metal/metal oxide nanomaterials have also been applied in pesticides detection, which can bind with target molecules efficiently and stably, and generate signals with different colors that can be detected by naked eyes or detectors.^[^ ^35^ ^]^ As a result, metal‐based nanomaterials with the effect of pathogenic microorganism inactivation and pesticides detection open a door for agriculture science. Besides, other nano materials including carbon‐based nanomaterials,^[^ ^36^ ^]^ enzyme‐like optical sensor,^[^ ^37^, ^38^ ^]^ quantum dots (QDs),^[^ ^39^ ^]^ and silicon carbide NPs^[^ ^40^ ^]^ will be contained in detail.

In this review, we overview the recent developments of advanced eco‐friendly nanoplatforms in the agricultural field, with an emphasis on the synthesis, mechanisms, and their applications in on‐site detection of agricultural chemicals and mycotoxins, control of plant pathogens, delivery of agricultural chemicals for pest control, and regulation of crops growth. Additionally, the advantages and potentials of eco‐friendly nanoplatforms in agricultural applications are covered through the investigation of examples reported in recent literatures. Finally, the developing prospects and challenges faced by nanoplatforms in agricultural applications are discussed comprehensively. In comparison with other prominent reviews related to the applications of nanoplatforms in the agricultural field, this review focuses on selecting outstanding researches of nanoplatforms for on‐site monitoring of pesticides and mycotoxins, controlling crops diseases in an eco‐friendly way, which will provide a flexible method for agricultural researchers to test crops safety in real‐time without polluting the environment. We introduce the development of nanoplatforms mainly in a chronological order in each section, which hopefully will give readers a clear picture of the development history and future development direction of nanoplatforms.

## 2 Eco‐Friendly Nanoplatforms for Pesticides Detection

Pesticides are generally used in the process of crops production to manage plant disease that may damage crops during production, storage or transport.^[^ ^41^ ^]^ However, pesticides residues can also contaminate agricultural products and induce potential health problems such as cancer and brain diseases.^[^ ^42^ ^]^ A case‐control study of non‐Hodgkin's lymphoma (NHL) in Kansas found that NHL was associated with the use of herbicides, and the relative risk of NHL increased significantly with the dose and the exposure time of herbicides.^[^ ^43^ ^]^ Glyphosate, as one of the most highly effective and broad‐spectrum pesticides, has been commonly used since it was commercially introduced in 1974, with an annual output of 700 thousand tons.^[^ ^35^, ^44^ ^]^ Recent reports indicate that glyphosate can accumulate in the soil for a long time due to the high affinity between phosphonate groups of glyphosate and iron oxides in the soil.^[^ ^45^ ^]^ Therefore, it is of great significance to pay attention to the fate and toxicity of pesticides. Currently, a majority of the reported methods for the detection of pesticides are based on chromatography and capillary electrophoresis, exhibiting the advantages of high sensitivity and strong stability.^[^ ^46^ ^]^ However, these traditional methods are difficult to adopt due to the high cost of testing equipment, complicated pretreatment of samples, long analysis time, and tedious sample collection and preparation. Therefore, the development of rapid, convenient, and efficient technology for on‐site pesticides detection is of strong demand and great value. In this section, we will introduce the nanoplatforms for on‐site monitoring of pesticides (**Table** **1**). These detection strategies will provide a flexible method for agricultural researchers to point‐of‐care test agricultural chemicals in crops without sample pretreatment.

**Table 1:** Eco‐friendly nanoplatforms for agricultural chemicals detection

Nanoplatform	Targeting	Detection method	Detection range	LOD	Ref.
SERS active tape	4‐Mercaptopyridine Parathion‐methyl Thiram Chlorpyrifos	SERS	10^−5^–10^−9^ – – –	1 nmol L^−1^ 2.60 ng cm^−2^ 0.24 ng cm^−2^ 3.51 ng cm^−2^	^[^ ^47^ ^]^
AgNP@AgNW PDMS	Thiram Malachite green	SERS	10^−10^−10^−5^ moL L^−1^	10^−10^ mol L^−1^ 10^−11^ mol L^−1^	^[^ ^48^ ^]^
AgNPs/GO paper	Thiram Thiabendazole Parathion‐methyl	SERS	8 × 10^−10^–8 × 10^−8^ g cm^−2^ 8.5 × 10^−8^–8.5 × 10^−6^ g cm^−2^ 2.2 × 10^−8^–2.2 × 10^−6^ g cm^−2^	0.26 ng cm^−2^ 28 ng cm^−2^ 7.4 ng cm^−2^	^[^ ^49^ ^]^
Au@Ag NCs	Thiram	SERS	0.24–12 mg kg^−1^	0.148 mg kg^−1^	^[^ ^50^ ^]^
Ag/NC substrate	Thiram Thiabendazole	SERS	– –	0.5 ng cm^−2^ 5 ng cm^−2^	^[^ ^51^ ^]^
AuNP‐Cys	Glyphosate	Colorimetric method SERS	≥ 0.001 mg L^−1^ 0.001–1000 mg L^−1^	– 0.026 mg L^−1^	^[^ ^52^ ^]^
SiCNPs@MWCNTs‐CS	Parathion	CV and DPV	–	20 ng mL^−1^	^[^ ^40^ ^]^
Nanoceria	Methyl‐paraoxon (MP)	Colorimetric method UV–vis spectroscopic method	2.1–21 µmol L^−1^ 0.42–42µmol L^−1^	0.42 µmol L^−1^ 0.42 µmol L^−1^	^[^ ^37^ ^]^
ACC‐HNFs	Paraoxon	CV Colorimetric method	6.0 × 10^−6^–0.6 ng mL^−1^ 0.01–100 ng mL^−1^	6.0 fg mL^−1^ 10 fg mL^−1^	^[^ ^38^ ^]^
AuNPs/PVC film	Thiram Parathion‐methyl	SERS	– –	10 ng cm^−2^ –	^[^ ^53^ ^]^
Ni–(OH)_2_ NSs	Acetochlor Fenpropathrin	Two‐way LFI with smartphone readout	1.0–150.0 ng mL^−1^ 1.0–150.0 ng mL^−1^	0.63 ng mL^−1^ 0.24 ng mL^−1^	^[^ ^54^ ^]^
ZnONPs (500 °C) ZnONPs (550 °C)	Aldicarb	Photoinduced electron transfer route	250 pmol L^–1^–2 nmol L^−1^ 250 pmol L^–1^–5 nmol L^−1^	250 pmol L^−1^ 250 pmol L^−1^	^[^ ^55^ ^]^
AuNPs	Prothioconazole	Colorimetric method	1.33–19.99 µg L^−1^	0.38 µg L^−1^	^[^ ^56^ ^]^

**Figure 2:**
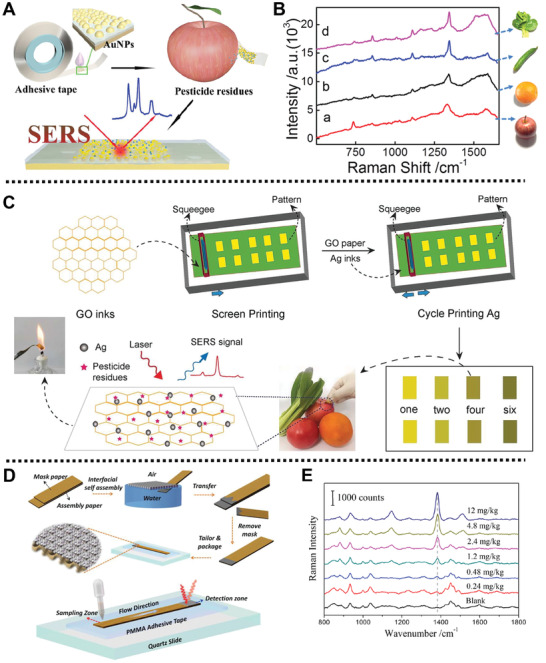
A) Schematic diagram of the preparation of SERS tape and its application of sensing pyridine and sulfur pesticides from apple peel by SERS analysis. B) Raman spectra of parathion‐methyl peeled from the surfaces of different fruits and vegetables using SERS tape. Reproduced with permission.^[^ ^47^ ^]^ Copyright 2016, American Chemical Society. C) Schematic description of the preparation of SERS swabs and its application for the detection of pesticides residues in garden stuff. Reproduced with permission.^[^ ^49^ ^]^ Copyright 2018, Royal Society of Chemistry. D) Schematic representation for the fabrication process of the lab‐on‐paper SERS platform and the SERS on‐site detection of thiram in the soil. E) Raman spectra of thiram at different concentrations in the soil using SERS platform on paper. Reproduced with permission.^[^ ^50^ ^]^ Copyright 2019, Wiley‐VCH Verlag GmbH & Co. KGaA, Weinheim.

To date, surface enhanced Raman spectroscopy (SERS) technique as a promising approach has been applied in different fields, such as, life sciences,^[^ ^57^ ^]^ biomedicine,^[^ ^58^ ^]^ homeland security,^[^ ^59^ ^]^ and environmental science,^[^ ^60^ ^]^ and it is capable of remarkably enhancing cross sections of Raman scattering and expanding the characteristic vibrational fingerprints of molecules for the detection of pesticides residues even at low concentration.^[^ ^57^ ^]^ Recently, NPs have been introduced into non‐traditional SERS substrates to overcome the drawbacks of silicon wafers and glass‐based substrates.^[^ ^49^ ^]^ Notably, SERS substrates functionalized by metal/metal oxide NPs, including AgNPs, AuNPs, and ZnONPs, are used as Raman signal amplifiers for the detection of agricultural chemicals through observing the color or fluorescence changes, providing a sensitive and convenient approach for visual and on‐site detection of agricultural chemicals.^[^ ^52^ ^]^ For instance, a highly sensitive multi‐pesticides sensing platform combined with AuNPs using an adhesive SERS substrate was reported by Guo and co‐workers for the extraction and detection of pyridine and sulfur pesticides by direct “paste and peel off” method (**Figure** **2**A).^[^ ^47^ ^]^ AuNPs were adhered onto the surface of “SERS double‐sided tape” via a drop‐dry approach. The as‐prepared SERS active tape was applied to detect 4‐mercaptopyridine with a linear range of 10^−5^ to 10^−9^ mol L^−1^. Moreover, experimental results showed that the SERS active tape had the ability of qualitative detection of pesticides residues on a series of garden stuff with various size and surface roughness, and exhibited outstanding enhanced SERS signals (Figure 2B). Furthermore, such platform could detect parathion‐methyl, thiram, and chlorpyrifos on cucumber, achieving a limit of detection (LOD) of 2.60, 0.24 , and 3.51 ng cm^−2^, respectively.

Analogously, Gao and co‐workers reported a three‐dimension (3D) detection platform based on SERS method, namely AgNP@AgNW PDMS, for the rapid on‐site detection of thiram and malachite green residues.^[^ ^48^ ^]^ AgNP@AgNW PDMS substrate consisting of AgNPs@AgNW as rich SERS “hot spots” and polydimethylsiloxane (PDMS) as support provided an effective adsorption surface for thiram and green residues in SERS detection, with a low LOD of 10^−10^ and 10^−11^ mol L^−1^, respectively. Besides, after spraying thiram onto the surface of spathiphyllum leaves, the samples on the leaves were adhered, peeled, and detected by AgNP@AgNW PDMS substrates, and the detection results were consistent with the concentration of the sprayed pesticides.

Subsequently, Fodjo and co‐workers prepared an ultrasensitive detection platform based on AgNPs and graphene oxide (GO) for the detection of pesticides residues in fruits and vegetables (Figure 2C).^[^ ^49^ ^]^ In this system, AgNPs and GO were printed on the cellulose paper substrate through a screen‐printing technique to produce AgNPs/GO paper. Experimental results showed that AgNPs/GO paper had excellent adsorption capacity towards thiram (620.5 mg cm^−2^) duo to the *π*–*π* interactions and electrostatic interactions between GO and pesticides. Besides, AgNPs/GO paper could detect parathion‐methyl, thiram, and thiabendazole quantitatively and qualitatively using SERS via swabbing the peels of the fruits and vegetables. This work provides a rapid and accurate approach to detect multi‐pesticides residues from food.

Wang and co‐workers designed a detection platform based on self‐assembled Au@Ag nanocube (Au@Ag NCs) using a lab‐on‐paper SERS strategy for on‐site detection of thiram residues in the soil (Figure 2D).^[^ ^50^ ^]^ Au@Ag NCs with the average size of 53 nm were deposited onto filter paper via liquid–liquid interface self‐assembly (LLISA) technology to form a paper‐based SERS substrate with the assistance of a mask paper. The experimental results showed that the paper‐based SERS substrate exhibited outstanding quantitative ability to detect thiram in the soil, reaching a LOD of 0.148 mg kg^−1^ (Figure 2E).

**Figure 3:**
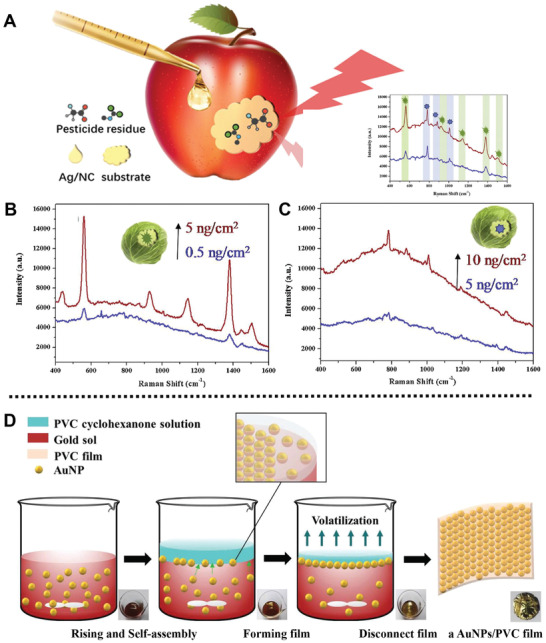
A) Schematic illustration of rapid SERS on‐site detection of pesticides residue from apple. B,C) Schematic description of the SERS on‐site detection of thiram and TBZ on cabbage surface, respectively. Reproduced with permission.^[^ ^51^ ^]^ Copyright 2019, Elsevier. D) Schematic representation for the preparation process of a AuNPs/PVC film. Reproduced with permission.^[^ ^53^ ^]^ Copyright 2010, Royal Society of Chemistry.

Interestingly, a jellylike platform based on nanocellulose and AgNPs was prepared by Huang and co‐workers for rapid on‐site detection of pesticides in fruits/vegetables (**Figure** **3**A).^[^ ^51^ ^]^ AgNPs were coated onto the surface of nanocellulose by a soaking method to form the Ag/NC substrate. In this case, the Ag/NC substrate with sticky textures and moldable property could directly smeared and adhered on the peels of apples and cabbages for effectively on‐site detection of thiram and thiabendazole by SERS strategy, with low detectable level of 0.5 and 5 ng cm^−2^, respectively (Figure 3B,C).

For another instance, a cysteamine‐modified AuNPs (AuNPs‐Cys) nanoplatform based on colorimetry and SERS detection strategy was designed by He and co‐workers for glyphosate targeting on spinach leaves.^[^ ^52^ ^]^ AuNPs‐Cys showed strong affinities to glyphosate via electrostatic interactions, and the cross‐linking within AuNPs could also capture the glyphosate molecules. The mechanism of this colorimetric‐SERS detection strategy was based on the intuitive color changes of AuNP‐Cys solution before and after interacting with the glyphosate molecules on the tissue surface of spinach leaves to realize rapid monitoring of glyphosate, reaching a LOD of 0.026 mg L^−1^. Moreover, experimental results showed that with the increasing concentrations of glyphosate on the spinach leaves, the color of AuNPs‐Cys solution changed obviously, which presents a convenient method to monitor glyphosate content on crops.

In 2019, Wang and co‐workers reported a paintable nanomaterial ink using silicon carbide NPs (SiC NPs) and multi‐walled carbon nanotubes (MWCNTs) for on‐site tracing parathion residues on vegetables.^[^ ^40^ ^]^ After dispersing SiC NPs and MWCNTs, the obtained ink was further stabilized by chitosan fixing glue to form chitosan coated electrode ink (SiCNPs@MWCNTs‐CS), which showed excellent electrochemical response to parathion, reaching a LOD of 20 ng mL^−1^. Moreover, the electrode ink was used to detect parathion in real agricultural products of Chinese cabbage, cucumber, and sweet potato leaf, and the recoveries were 76.0–96.2%. This electrode ink exhibited potential applications in the detection of pesticides residues on vegetables and showed good stability even after storage for 21 d.

As a catalytic material, nanoceria has excellent enzyme‐like activity to hydrolyze certain chemical component, and the whole hydrolysis process is accompanied by the color change of the hydrolysate, which can be used to on‐site detection of pesticides. For example, Li and co‐workers used nanoceria on the basis of colorimetric and spectroscopic strategies for on‐site detection of organophosphorus pesticides in Semen nelumbinis, Semen Armeniacae Amarum, and Rhizoma *Dioscoreae*.^[^ ^37^ ^]^ In this case, nanoceria with enzyme‐like activity was prepared by the reaction of cerium chloride, hydrogen peroxide, and ammonium hydroxide. The detection mechanism of the prepared nanoceria for methyl‐paraoxon (MP) in real samples was to hydrolyze MP into para‐nitrophenol, which was accompanied by the changes in colors and UV–vis absorbance of solutions. Meanwhile, the dual‐mode detection results showed that nanoceria exhibited good detection abilities for MP in Semen nelumbinis, Semen Armeniacae Amarum, and Rhizoma *Dioscoreae* and no MP was detected, which was consistent with the results of Gas Chromatography Mass Spectrometry. This work proposes a new dual‐mode detection strategy for on‐site detection of organophosphorus pesticides, which conquers the limitations of natural enzymes in terms of instability.

In 2019, a tandem catalysis‐driven paper‐based biosensor using enzyme‐inorganic hybrid nanoflowers for targeting organophosphorus pesticides was fabricated by Lu and co‐workers by a one‐pot protein biomineralization strategy.^[^ ^38^ ^]^ Cu_3_(PO_4_)_2_·3H_2_O as inorganic scaffold, reacted with acetylcholine esterase (AChE) and choline oxidase (ChO) by the coordination bond between Cu^2+^ and nitrogen atoms of amide groups in AChE and ChO, to achieve the artificial enzyme cascade biosensor called AChE/ChO/Cu_3_(PO_4_)_2_‐HNFs (ACC‐HNFs). Generally, ACC‐HNFs with AChE and ChO activities could catalyze the hydrolysis of ACh to generate H_2_O_2_ and further catalyze the 3,3′,5,5′‐tetramethylbenzidine (TMB) into colored oxTMB. When paraoxon was introduced, the AChE activity of ACC‐HNFs was directly suppressed, which caused the absorbance change of the system, thus realizing the detection of organophosphorus pesticides. The real sample analysis results showed the paper‐based dual‐readout biosensor had great reliability and applicability for monitoring paraoxon residues in water, rice, and apples. This lab‐on‐paper platform possessed dual‐modal analytical method, including spectroscopic strategy and colorimetric strategy, for on‐site detection of paraoxon pesticides with the linear range from 6.0 × 10^−6^ to 0.6 ng mL^−1^, and reaching a low LOD of 6.0 fg mL^−1^.

Interestingly, Zheng and co‐workers fabricated a AuNPs/polyvinyl chloride (PVC) detection film as SERS substrates, which could be directly wrapped on the surface of apples for on‐site detection of multi‐pesticides residues.^[^ ^53^ ^]^ After synthesis of the AuNPs by one‐step reduction method, PVC was introduced to induce the interfacial self‐assembly of AuNPs, and then the assembled AuNPs were half‐embedded into the PVC matrix for immobilization to obtain the AuNPs/PVC film (Figure 3D). In the actual application test, AuNPs/PVC film with outstanding flexibility and stability could be directly swabbed onto the surface of the apples contaminated by thiram, which showed an increasing prominent SERS peak at 1378 cm^−1^ with the increasing concentration of thiram with a LOD of 10 ng cm^−2^. In addition, with the help of a portable Raman spectrometer, field test results revealed that a prominent Raman signal of thiram and parathion‐methyl was detected from the apples contaminated by thiram and parathion‐methyl using the prepared AuNPs/PVC film, providing a feasible method for on‐site detection of pesticides. Besides, the AuNPs/PVC film could be regenerated after cleaning with NaBH_4_ solution, demonstrating its superior reproducibility and stability.

More interestingly, two‐dimension (2D) Pt–Ni(OH)_2_ nanosheets (NSs) based on two‐way lateral flow immunoassay (LFI) were designed by Du and co‐workers for simultaneous detection of acetochlor and fenpropathrin by a smartphone readout.^[^ ^54^ ^]^ Ni(OH)_2_ NSs synthesized by a microwave method were used to load Pt NPs and decorate with anti‐acetochlor antibody or anti‐fenpropathrin antibody in turn to form antibody modified Pt–Ni(OH)_2_ NSs detection device with signal amplification. Immunoassay experiment results indicated that this detection device exhibited high peroxidase‐like activity in the detection of acetochlor and fenpropathrin, showing LOD of 0.63 and 0.24 ng mL^−1^, respectively. In addition, real food samples assay showed the proposed system provided an accurate on‐site monitoring strategy for simultaneous detection of herbicide and insecticide.

Recently, Solanki and co‐workers prepared a novel optical sensor based on ZnONPs with the average size of 70–150 nm for on‐site detection of aldicarb.^[^ ^55^ ^]^ After directly calcining zinc acetate at various temperatures (450, 500, and 550 °C), ZnONPs were prepared and employed for sensing aldicarb by a photoinduced electron transfer route. A series of experimental results proved that the synthesized ZnONPs (500 °C) and ZnONPs (550 °C) could monitor aldicarb in the range of 250–2 nmol L^−1^ and 250–5 nmol L^−1^, respectively, and LOD for aldicarb was 250 pmol L^−1^. This study paves a new way to develop a commercial device for on‐site sensing pesticides in food samples.

Yuan and co‐workers reported a simple citrate‐capped AuNPs platform based on colorimetric detection method for on‐site monitoring fungicides.^[^ ^56^ ^]^ The citrate‐capped AuNPs probe were prepared by citrate reduction method. Prothioconazole could be facilely combined onto the surface of AuNPs through Au—S bonds, and the hydrogen bonds among the prothioconazole molecules could induce the aggregation of citrate‐capped AuNPs, which followed by the color changes of the solution, and could be monitored by colorimetric method. Besides, various concentrations of prothioconazole (from 1.33 to 19.99 µg L^−1^) treated with the citrate‐capped AuNPs showed a concentration‐dependent color change, reaching a LOD of 0.38 µg L^−1^. Moreover, recovery experimental results showed that the average recoveries of prothioconazole were among 83.6–99.4% in rice field water samples, indicating high detection accuracy of prothioconazole using citrate‐capped AuNPs.

## 3 Eco‐Friendly Nanoplatforms for Mycotoxins Detection

Mycotoxins, a kind of pathogenic microorganism, belong to the secondary metabolites produced by fungi species, when agricultural crops are stored under unfavorable temperature and humidity conditions. As a type of mycotoxins that can contaminate various agricultural products, aflatoxins (AFs) have been listed in the Group I carcinogen by the International Agency for Research in Cancer.^[^ ^61^ ^]^ Besides, other mycotoxins, such as fumonisin B_1_ (FB_1_) produced by *Fusarium* species, ochratoxin produced by several *Aspergillus* and *Penicillium* species, have also attracted widespread attention.^[^ ^62^ ^]^ Contaminated food is difficult to distinguish, but once contaminated, it may induce a series of diseases, including neuronal damage, and Huntington disease symptoms.^[^ ^63^ ^]^ Unfortunately, the high physicochemical stabilities of most mycotoxins enable them to survive during the modern food manufacture process. Therefore, monitoring of mycotoxins in agricultural products is crucial to ensure food safety. Currently, for the effective control of mycotoxins, the prevailing detection methods mainly focus on chromatography, immunochemistry, and gene techniques, which are proven to be accurate and reliable.^[^ ^64^ ^]^ However, some drawbacks in real‐time detection applications, such as professional laboratory conditions, complex sample preparation, and time‐consuming, make them unsuitable for on‐site rapid screening and detection of mycotoxins contaminations. Therefore, the development of convenient, cost‐effective mycotoxins detection platforms is of great significance to global food safety. In this part, we will introduce the applications and advantages of the nanoplatforms for on‐site detection of mycotoxins in crops (**Table** **2**).

**Table 2:** Eco‐friendly nanoplatforms for mycotoxins detection

Nanoplatform	Targeting	Detection method	Detection range	LOD	Ref.
UCNP‐based LRET aptasensor	OTA	LRET aptasensor	0.1–1000 ng mL^−1^	0.098 ng mL^−1^	^[^ ^65^ ^]^
Urease‐induced AuNFs pELISA	OTA	Colorimetric pELISA	5.0–640 pg mL^−1^	8.205 pg mL^−1^	^[^ ^66^ ^]^
AuE‐dc‐pELISA	AFB_1_	Colorimetric method	3.1–150 pg mL^−1^	4.0 pg mL^−1^	^[^ ^67^ ^]^
rMoS_2_‐Au aptasensor	ZEN FB_1_	DPV	1 × 10^−3^–10 ng mL^−1^ 1 × 10^−3^–10^2^ ng mL^−1^	5 × 10^−4^ ng mL^−1^ 5 × 10^−4^ ng mL^−1^	^[^ ^28^ ^]^
Tricolor QB‐based mICA	OTA FB_1_ ZEN	mICA method	– – –	5 ng mL^−1^ 20 ng mL^−1^ 10 ng mL^−1^	^[^ ^39^ ^]^
AuNP‐based multiplex ICTS nanosensor	FB_1_ ZEN OTA AFB_1_	Colorimetric method	4–80 ng mL^−1^ 0.8–40 ng mL^−1^ 0.2–2 ng mL^−1^ 0.1–1.25 ng mL^−1^	3.27 ng mL^−1^ 0.70 ng mL^−1^ 0.10 ng mL^−1^ 0.06 ng mL^−1^	^[^ ^68^ ^]^

Compared with the traditional methods of mycotoxins detection using common materials, nano materials modified aptamers, double‐stranded DNA or single‐stranded RNA can bind to target molecules with high affinity and selectivity.^[^ ^69^ ^]^ As molecular probes, aptamers are easy to be synthesized, modified, and immobilized on the surface of nanomaterials for simultaneous assay of multiplex mycotoxins, indicating superior performances.^[^ ^70^ ^]^ On this basis, in 2017, Kim and co‐workers prepared a luminescence resonance energy transfer (LRET) aptasensor based on Mn^2+^‐doped NaYF_4_:Yb^3+^,Er^3+^ UCNPs for rapid and sensitive detection of mycotoxins.^[^ ^65^ ^]^ Black hole quencher 3 acceptor (BHQ3) labeled ochratoxin A (OTA) aptamer was immobilized onto the surface of carboxylated‐PEG modified Mn^2+^‐doped NaYF_4_:Yb^3+^,Er^3+^ UCNPs donor to form the final LRET aptasensor. Due to the overlap between the emission of UCNPs and the absorption of BHQ3, and the shortening of the aptamer chain length caused by the combination of the target molecule and the aptamer, the upconversion luminescence of Mn^2+^‐doped UCNPs donor under laser irradiation at 980 nm was quenched. Moreover, the shorter distance between BHQ3 and UCNPs, the quenching efficiency was larger. Mycotoxins assay results showed the BHQ3 labeled aptamer folded on the surface of the UCNPs to form the aptamer‐target complex, when mycotoxins contaminated food samples were added into the solution containing UCNPs nanoprobe. Under the optimized conditions, the UCNP‐based LRET aptasensor could on‐site detect spiked OTA‐contaminated food samples within 10 min with the LOD of 0.098 ng mL^−1^.

**Figure 4:**
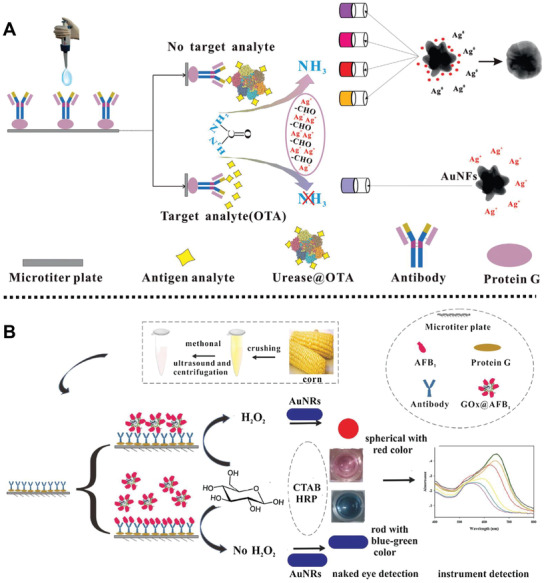
A) Schematic illustration of the urease‐induced AuNFs pELISA for on‐site detection of OTA cereal samples. Reproduced with permission.^[^ ^66^ ^]^ Copyright 2018, Elsevier. B) Schematic diagram of the preparation of AuE‐dc‐pELISA for the detection of AFB_1_ in corn samples. Reproduced with permission.^[^ ^67^ ^]^ Copyright 2018, Elsevier.

Interestingly, Xiong and co‐workers reported an ultrasensitive nanoplatform based on gold nanoflowers (AuNFs) for on‐site detection of OTA in cereal samples using urease‐induced metallization plasmonic enzyme‐linked immunosorbent assay (pELISA) (**Figure** **4**A).^[^ ^66^ ^]^ AuNFs as colorimetric indicators possessed the surface plasmon resonance properties, and could produce obvious color readout when their shape changed. OTA‐labeled urease (urease@OTA) was synthesized by an esterification method and coupled with anti‐OTA monoclonal antibodies (mAbs), which would hydrolyze urea to generate ammonia. Then, the amount of Ag^+^ was reduced by glucose in presence of ammonia to form a shell onto the external of AuNFs, leading to the changes of flower‐like AuNFs into spherical ones, which in turn induced the changes of surface plasmon resonance peak and solution color (from blue to brownish‐yellow). Experimental results showed that the colorimetric pELISA exhibited high sensitivity and specificity to OTA, showing an ultrasensitive LOD of 8.205 pg mL^−1^.

In 2018, a high sensitivity sensing platform based on gold nanorods (AuNRs) was reported by Xiong and co‐workers for simultaneous quantitative and qualitative detection of aflatoxin B_1_ (AFB_1_) using plasmonic enzyme‐linked immunosorbent assay (pELISA) (Figure 4B).^[^ ^67^ ^]^ In this case, AuNR‐etching‐based direct competitive pELISA (AuE‐dc‐pELISA) detection platform was composed of AuNRs as colorimetric substrate, AFB_1_‐labeled glucose oxidase as competing antigen, glucose, and horseradish peroxidase. Experimental results showed that the color of AuE‐dc‐pELISA solution changed from pink to bluish‐green when the concentration of AFB_1_ was above 12.5 pg mL^−1^ in corn samples, which was ascribed to the competitive binding between AFB_1_ and anti‐AFB_1_ mAb, achieving a low LOD of 4.0 pg mL^−1^ for AFB_1_.

**Figure 5:**
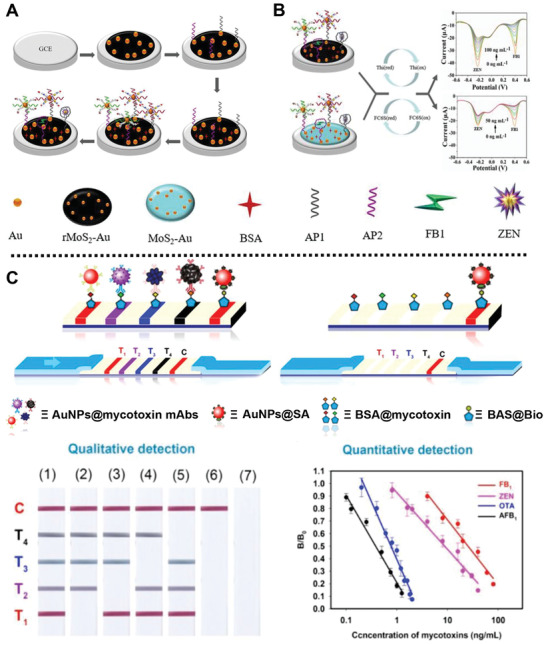
A) Schematic description of the fabrication process of the electrochemical sensor for screening ZEN and FB_1_, and B) the detection mechanism of the prepared platform. Reproduced with permission.^[^ ^28^ ^]^ Copyright 2020, Elsevier. C) Schematic representation for colored AuNP‐based incorporation ICTS probes with four T lines for the detection of FB_1_, ZEN, OTA, and AFB_1_, and the T/C value curves of the AuNP‐based probes for quantitative test of the four mycotoxins. Reproduced with permission.^[^ ^68^ ^]^ Copyright 2020, Elsevier.

Recently, a dual‐target electrochemical aptasensor based on aptamer, co‐reduced MoS_2_, and AuNPs was designed by Zhao and co‐workers for screening zearalenone (ZEN) and FB_1_ (**Figure** **5**A).^[^ ^28^ ^]^ AuNPs were introduced into the MoS_2_ via Au—S bonds to develop rMoS_2_‐Au hybrid composites, followed by the modification of aptamers of the ZEN and FB_1_ to obtain the aptasensor. Moreover, after modification of AuNPs by complementary DNA sequences (CP1 and CP2) of the aptamer, thionine (Thi), and 6‐(ferrocenyl) hexanethiol (FC6S), CP1‐Au‐Thi and CP2‐Au‐FC6S were prepared and could be bound with aptasensor through corresponding encoding positions. The cyclic voltammetry (CV) and differential pulse voltammetry (DPV) assays showed that the peak current intensities could gradually increase, when ZEN and FB_1_ molecules combined with their corresponding aptamer to replace CP1‐Au‐Thi and CP2‐Au‐FC6S (Figure 5B), with a LOD of 5 × 10^−4^ ng mL^−1^. Moreover, the recovery rates for ZEN and FB_1_ in maize samples were from 95.9 to 105.2%, and from 93.8 to 102.4%, respectively, which exhibited the feasibility of the designed aptasensor for on‐site monitoring ZEN and FB_1_ in real samples.

For another instance, Xiong and co‐workers reported three colored quantum dot (QD) nanobeads (QBs) for simultaneous on‐site qualitative detection of ZEN, OTA, and FB_1_ in corn samples using multiplex immunochromatographic assay (mICA).^[^ ^39^ ^]^ The three colored QB‐based mICA platform was prepared by encapsulating CdSe/ZnS QDs into polymer nanobeads, and then combined with the mAbs of the anti‐ZEN, anti‐OTA, or anti‐FB_1_, reaching low LOD of 5, 20, and 10 ng mL^−1^ for OTA, FB_1_, and ZEN, respectively, which provides a new method for the detection of multiple mycotoxins to improve food safety. Similarly, their group reported a simple, sensitive immunochromatographic test strip (ICTS) nanosensor based on four colored AuNPs for simultaneous on‐site targeting four mycotoxins, i.e., FB_1_, ZEN, OTA, and AFB_1_ in corn samples (Figure 5C).^[^ ^68^ ^]^ In this system, the colored AuNPs synthesized by morphology‐/size‐engineering methods were used as colored labels and signal transducers and modified by four corresponding mycotoxins mAbs to form four different AuNP@mAb probes immobilized onto the surface of nitrocellulose membranes. The mechanism of this nanosensor for mycotoxins was based on the color changes of the AuNPs@mAb solutions before and after the addition of the samples containing target mycotoxins, which was due to the competitive binding between target mycotoxins and competing antigens that had been bound to the AuNPs@mAb. Experimental results demonstrated the designed multicolor ICTS nanosensor had great detection sensitivity for simultaneously monitoring FB_1_, ZEN, OTA, and AFB_1_ with LOD of 3.27, 0.70, 0.10, and 0.06 ng mL^−1^, respectively.

## 4 Eco‐Friendly Nanoplatforms for Phytopathogen Inactivation

Based on the important impact on science/economy, a top 10 fungal plant pathogen list in *Molecular Plant Pathology* was reported in 2012 by Foster and co‐workers.^[^ ^71^ ^]^ *Magnaporthe oryzae*, *Botrytis cinerea* (*B. cinerea*), and *Puccinia* spp. were listed in the top three. *B. cinerea* as a typical necrotroph plant pathogen can produce gray powdery mould and infect more than 200 crop hosts worldwide, showing most destructive on mature tissues of dicotyledonous plants.^[^ ^72^ ^]^ Moreover, *B. cinerea* has developed resistance to many classes of conventional broad‐spectrum fungicides due to its genetic plasticity, and the use of synthetic fungicides has been proved to be a potential threat to human beings and the eco‐environment.^[^ ^73^ ^]^ So far, similar to *B. cinerea*, various multidrug resistance pathogens have become one of the main obstacles affecting crop growth. Breeding of resistant varieties of crops is an extremely effective means for disease management, whereas this method is time consuming and labor intensive. Therefore, it is increasingly essential to develop high performance fungicides to control phytopathogenic pathogens and keep less harmful to human beings and the environment.

At present, numerous eco‐friendly nanoplatforms to manage phytopathogenic pathogens in the agricultural field have been explored, such as MSNs‐based delivery system, GO‐based nanocomposite, Ag‐based nanoplatforms, and metal oxide NPs, which provide a safer way than synthetic pesticides to inactivate pathogens. In this part, we will introduce the synthesis and applications of eco‐friendly nanoplatforms against plant pathogens (**Table** **3**).

**Table 3:** Eco‐friendly nanoplatforms for phytopathogen inactivation

Nanoplatform	Pathogenic pathogen	Antibacterial mechanism	Plant	Ref.
AgTNTs	*B. cinerea*	Photocatalytic inactivation, ROS, and physical injury	–	^[^ ^74^ ^]^
TOL‐AgNPs	*X. axonopodis*, *P. syringae*	AgNPs	–	^[^ ^75^ ^]^
MgONPs	*R. solanacearum*	Physical injury and ROS	Tobacco plants	^[^ ^76^ ^]^
MgONPs	*P. nicotianae*, *T. basicola*	Physical injury and ROS	Tobacco plants	^[^ ^77^ ^]^
N, F co‐doped TiO_2_ NPs	*F. oxysporum*	Photocataly generate ROS	Tomato plants, fruits	^[^ ^78^ ^]^
Py@MSNs‐HTCC	*P. asparagi*	Release Py	–	^[^ ^79^ ^]^
AZOX‐loaded MSN‐CMCS	*P. infestans*	Release AZOX	Tomato plants	^[^ ^80^ ^]^
Lactose‐capped CNAD (AIT or Ajwain)‐loaded MSNs	*P. syringae* pv*. pisi*, *P. fluorescens*, *P. carotovorum* subsp. *carotovorum*	Release CNAD, AIT, and Ajwain	Peas	^[^ ^81^ ^]^
Py@F‐DSH‐MSNs	*P. asparagi*, *P. xanthii*	Release Py	Cucumber leaves	^[^ ^82^ ^]^
ZnONPs and SiO_2_NPs	TMV	Physical injury, ROS, and the activation of the plants antioxidant system	Tobacco plants	^[^ ^83^ ^]^
AZOX@MSNs‐PDA‐Cu	*P. oryzae*, *P. xanthii*	Release AZOX	Cucumber plants	^[^ ^84^ ^]^
PRO‐MON‐CaC	*S. sclerotiorum*	Release PRO	Rapeseed plants	^[^ ^85^ ^]^
GO‐AgNPs	*F. graminearum*	Physical injury and ROS	Wheat leaves	^[^ ^86^ ^]^
Hy‐GO@PDA	*F. xysporum*	Release Hy	–	^[^ ^87^ ^]^
CMV2b‐BioClay PMMoVIR54‐BioClay	PMMoV, CMV	Formation of endogenous siRNAs	Cowpea, tobacco	^[^ ^88^ ^]^

For instance, three AgNPs‐functionalized titanate nanotubes (AgTNTs) were utilized by González and co‐workers for the effective photoinactivation of *B. cinerea*.^[^ ^74^ ^]^ Titanate nanotubes (TNTs) with 0.5%, 1%, and 3 wt% AgNPs loading exhibited 60%, 70%, and 100% photoinactivation abilities against *Escherichia coli* under light irradiation. Meanwhile, compared with 1% and 3% AgNPs‐loaded TNTs, 0.5% AgNPs‐loaded TNTs presented 100% inactivation capability against *B. cinerea* within 20 min under visible‐light irradiation, which was due to the high sensitivity of *B. cinerea* to the sharp morphology of TNTs. Scanning electron microscopy (SEM) and transmission electron microscopy (TEM) studies demonstrated that TNTs in AgTNTs could penetrate into the conidia from the external cell wall to the inside cell, and induce the fusion of intracellular vacuoles, thus causing the complete inactivation of *B. cinerea*. Besides, the generation of radical species under light irradiation and stress aerobic metabolism could also result in the inactivation of *B. cinerea*, with the viability loss of pathogenic fungus.

Saratale and co‐workers developed a smart nanoplatform based on AgNPs with an average diameter of 15 nm for the inactivation of two phytopathogens, *Xanthomonas axonopodis* pv. *citri* (*X. axonopodis*) and *Pseudomonas syringae* (*P. syringae*).^[^ ^75^ ^]^ TOL‐AgNPs mediated by the leaf extractive of medicinal plant *Taraxacum officinale* were synthesized via a green synthesis approach. The zones of inhibition (ZOI) of commercial AgNPs are 16.2 mm for *X. axonopodis* and 14.4 mm for *P. syringae*. In clear contrast, an enhanced ZOI was observed in TOL‐AgNPs group, which showed 21.4 and 18.1 mm for *X. axonopodis and P. syringae*, respectively. In addition, the minimum inhibitory concentration values of TOL‐AgNPs were 20 µg mL^−1^ for *X. axonopodis* and 25 µg mL^−1^ for *P. syringae*. However, the antibacterial mechanism of this system and its actual applications in the agricultural field were not involved.

**Figure 6:**
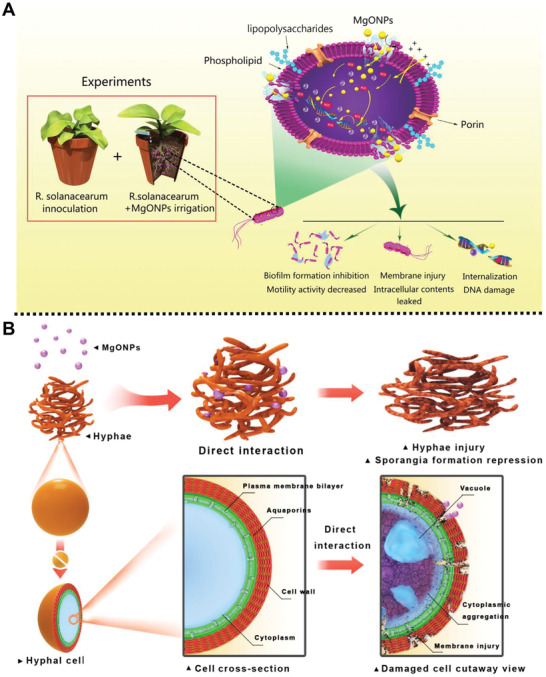
A) Schematic description of the toxicity mechanism of the preparation of MgONPs in response to phytopathogenic *R. solanacearum*. Reproduced with permission.^[^ ^76^ ^]^ Copyright 2018, Frontiers Media S.A. B) Schematic summarization of the interactions between the MgONPs and fungal pathogen conidia and mycelia. Reproduced with permission.^[^ ^77^ ^]^ Copyright 2018, Frontiers Media S.A.

In addition, an eco‐friendly bactericidal material based on magnesium oxide NPs (MgONPs) was prepared by Ding and co‐workers for the inhibition of phytopathogenic *Ralstonia solanacearum* (*R. solanacearum*) (**Figure** **6**A).^[^ ^76^ ^]^ Compared with bulk MgO, MgONPs exhibited excellent growth inhibition effects on *R. solanacearum* due to the high Brunauer–Emmett–Teller surface areas and small particle size of MgONPs. Experimental results indicated that MgONPs could penetrate bacterial cells of *R. solanacearum* and induce the generation of ROS, resulting in the leakage of the intracellular content and bacterial cells death. Moreover, greenhouse experimental studies demonstrated that MgONPs could reduce the bacterial wilt index of tobacco plants caused by *R. solanacearum*, which provided an efficient alternative to chemical pesticides for crops protection. Subsequently, their group studied the fungicidal activities of MgONPs and macroscale magnesium oxide (mMgO) against *Thielaviopsis basicola* (*T. basicola*) and *Phytophthora nicotianae* (*P. nicotianae*) (Figure 6B).^[^ ^77^ ^]^ MgONPs with an average diameter of 100 nm were prepared with the extractive of *Carica papaya* L. leaf and magnesium nitrate through a green synthesis process. Experimental studies demonstrated that MgONPs showed significant inhibitory effects on mycelial growth, conidiospore germination, and sporulation for the two fungi. Comparatively, mMgO showed a marked hyphae growth inhibition to *P. nicotianae* and a moderated growth inhibition to *T. basicola*. Both MgONPs and mMgO revealed concentration‐ and time‐dependent antifungal effects. Importantly, MgONPs exhibited significantly antifungal activity to *P. nicotianae* and *T. basicola* in tobacco seedlings in the pot experiments. This work paves a new road for the management of tobacco black shank and root black rot using magnesium‐based fungicides.

Recently, a visible light‐activated antifungal agent based on N and F co‐doped TiO_2_ NPs was fabricated by Jana and co‐workers for the treatment of *Fusarium oxysporum* (*F. oxysporum*).^[^ ^78^ ^]^ N and F co‐doped TiO_2_ NPs were coated with polyacrylate shell and fluorescein *o*‐methacrylate to obtain polymer‐coated doped NPs with hydrophilic surface, zwitterionic charge and tracking agent. Antifungal assay results showed that polymer‐coated doped NPs exhibited significant antifungal activity against fungal mycelia of *F. oxysporum* under visible light via the generation of ROS. Besides, polymer‐coated doped NPs possessed the completely inhibit effect to the growth of *F. oxysporum* in tomato fruit under visible light irradiation.

**Figure 7:**
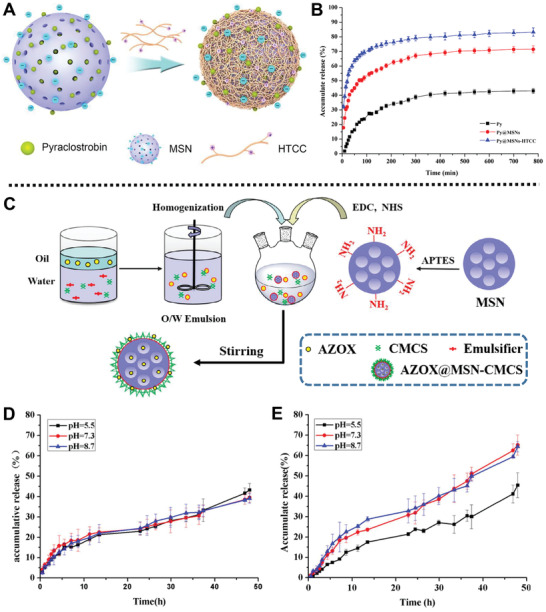
A) Schematic diagram of the fabrication process of the Py@MSNs‐HTCC platform. B) Release profiles of Py from Py@MSNs‐HTCC, Py@MSNs, and pyraclostrobin technical, respectively. Reproduced with permission.^[^ ^79^ ^]^ Copyright 2016, MDPI. C) Schematic description of the preparation process of the AZOX@MSN‐CMCS platform. Release profiles of AZOX from D) AZOX@MSN‐CMCS and E) AZOX@MSN at different pH values. Reproduced with permission.^[^ ^80^ ^]^ Copyright 2018, Elsevier.

In addition, stimuli‐responsive antimicrobial release nanoplatforms based on MSNs can be functionalized with multifunctional materials to achieve the controlled release of cargos. For instance, a fungicide delivery system based on MSNs for the management of *P. asparagi* was prepared by Huang and co‐workers (**Figure** **7**A).^[^ ^79^ ^]^ In this case, *N*‐(2‐Hydroxyl)propyl‐3‐trimethylammonium CS chloride (HTCC) was coated onto the surface of pyraclostrobin (Py)‐loaded MSNs by electrostatic interaction and hydrogen bond to form supramolecular complex Py@MSNs‐HTCC with a high loading capacity of 40.3%. Experimental results demonstrated that 72% Py could be released from Py@MSNs‐HTCC within 2 h, which exhibited a high burst release at initial time to satisfy the need for immediate treatment against *P. asparagi* (Figure 7B). Moreover, in vitro experiments demonstrated that Py@MSNs‐HTCC (10.0 mg L^−1^) and Py (20.0 mg L^−1^) performed almost the same inhibition activity for *P. asparagi* after 6 d of treatment, which indicated that Py@MSNs‐HTCC nanoplatform could enhance the utilization efficiency of Py.

A pH‐responsive emulsion‐based fungicide delivery system was designed by Huang and co‐workers for effective inhibition of the growth of tomato late blight *Phytophthora infestans* (*P. infestans*) (Figure 7C).^[^ ^80^ ^]^ In this system, amino group‐functionalized MSNs were loaded with azoxystrobin (AZOX) and coated with carboxymethyl chitosan (CMCS) simultaneously to form AZOX‐loaded MSN‐CMCS. Controlled release experiments showed that the release of AZOX from AZOX@MSN had no obvious difference under three pH conditions (pH 5.5, 7.3, and 8.7) (Figure 7D). However, a marked release (66%) of AZOX from the AZOX‐loaded MSN‐CMCS was observed at pH 7.3 and 8.7 after 48 h, which was ascribed to the stretching of CMCS chains under alkaline and neutral conditions (Figure 7E). Bioactivity experiments showed that AZOX‐loaded MSN‐CMCS (20.0 mg L^−1^) exhibited 65% fungicidal activity against *P. infestans*. Besides, confocal laser scanning microscopy images showed isothiocyanate labeled MSN‐CMCS could distribute throughout the whole tomato plants and the fungi body, which demonstrated that MSN‐CMCS could be used as stimuli‐responsive nanocarriers to deliver pesticides in tomato plants and fungi.

In 2018, Thompson and co‐workers fabricated an antimicrobials delivery system based on MSNs for the inhibition of *Pseudomonas syringae* pv. *Pisi* (*P. syringae* pv. *Pisi*), *Pseudomonas fluorescens* (*P. fluorescens*), and *Pectobacterium carotovorum* subsp. *carotovorum* (*P. carotovorum* subsp. *carotovorum*).^[^ ^81^ ^]^ Cinnamaldehyde (CNAD), allyl isothiocyanate (AIT), and Ajwain essential oils (EOs) as antimicrobials were loaded into MSNs, respectively, and then a lactose derivative was capped onto the surface of MSNs to form lactose‐capped CNAD (AIT or Ajwain)‐loaded MSNs. The bacterial viability studies showed lactose‐capped CNAD‐loaded MSNs exhibited the best bacterial elimination rate of >99.9%, >99.8%, and >95% for *P. fluorescens*, *P. syringae* pv. *Pisi*, and *P. carotovorum* subsp. *carotovorum*, respectively. Furthermore, the prepared lactose‐capped CNAD‐loaded MSNs could protect EOs from evaporation and degradation, exhibiting a longer‐lasting and enhanced antimicrobial activity compared with free EOs.

Subsequently, Huang and co‐workers prepared a smart fungicide delivery system based on fluorescent double‐shelled hollow MSNs (F‐DSH‐MSNs) for the inhibition of *P. asparagi* and *Pyricularia xanthii* (*P. xanthii*).^[^ ^82^ ^]^ In this platform, carbon dots (CDs) as luminescent agent were embedded in DLH‐MSNs to form F‐DSH‐MSNs after calcination treatment. Then, Py was loaded into the carrier to develop Py@F‐DSH‐MSNs with loading content of 28.5%. Experimental results showed the cumulative release of Py from the DSH‐MSNs reached 68% at pH 7.0 after 100 h, showing slow and sustained release behaviors. SEM and TEM studies showed that the prepared NPs could deliver fungicide into hyphae cell and induce the decrease of mitochondria and vacuoles in the mycelium. Besides, the preventability tests showed that *P. xanthii* treated with Py@F‐DSH‐MSNs and commercial Py exhibited almost similar preventive activity against cucumber powdery mildew, indicating good bioavailability and applicability of Py@F‐DSH‐MSNs.

**Figure 8:**
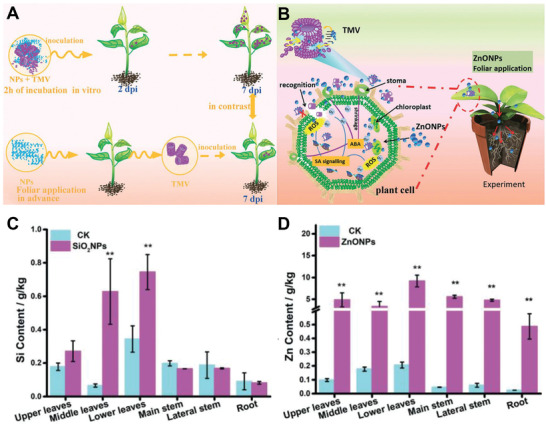
A) Schematic summarization for the process of NPs in response to TMV in tobacco plants. B) Mechanisms of antiviral activity of ZnONPs in *N. benthamiana* plants. Accumulation and distribution of C) Si^4+^ and D) Zn^2+^ in the different parts of plants treated with 100 µg mL^−1^ SiO_2_NPs and ZnONPs. Reproduced with permission.^[^ ^83^ ^]^ Copyright 2019, Royal Society of Chemistry.

Sun and co‐workers synthesized ZnONPs and SiO_2_NPs for the management of *Tobacco mosaic virus* (TMV) by directly deactivating TMV and activating plant immunity (**Figure** **8**A).^[^ ^83^ ^]^ TEM images studies showed the aggregation and fracture of TMV and the decrease of virus colonization on tobacco plants after treating the leaves of *Nicotiana benthamiana* (*N. benthamiana*) with the mixture of TMV and NPs for 2 d. ZnONPs and SiO_2_NPs could induce the physical injury of TMV shell proteins and improve the self‐defense of plants, thus realizing the management of TMV (Figure 8B). Significantly, in vivo studies showed that ZnONPs and SiO_2_NPs (100 µg mL^−1^) could remarkably inhibit the viral multiplication and enhance tobacco growth with less deformed leaves. Furthermore, experimental results confirmed that SiO_2_NPs and ZnONPs were absorbed and transported into the cells of the host plant, inducing the generation of ROS or the activation of the antioxidant system of plants (Figure 8C,D). Besides, plant growth response studies showed that the *N. benthamiana* plants treated with ZnONPs exhibited increased salicylic acid (SA) phytohormone synthesis and up‐regulated SA‐responsive resistance gene, which enhanced the self‐defense of plant against TMV and provided an eco‐friendly alternative to chemical antiviral drugs for controlling TMV.

In addition, a pH‐responsive nanoplatform based on MSNs was fabricated by Huang and co‐workers for the inhibition of *Pyricularia oryzae* (*P. oryzae*) and *P. xanthii*.^[^ ^84^ ^]^ MSNs as drug reservoirs were loaded with AZOX and modified with polydopamine (PDA) film as a gatekeeper, and then Cu^2+^ was chelated with the catechol and amines moieties of the PDA film to form AZOX@MSNs‐PDA‐Cu. The release assays showed that the accumulated release of AZOX at pH 5.8 and 8.6 was higher than that at neutral pH, which was ascribed to the strong competitive coordination between H^+^ and the nitrogen atoms of the PDA at acidic condition or OH^−^ and Cu^2+^ at alkaline condition. Experimental studies showed that AZOX@MSNs‐PDA exhibited high bioactivity against rice blast fungus (*P. oryzae*) and cucumber powdery mildew pathogen (*P. xanthii*). Besides, AZOX@MSNs‐PDA‐Cu possessed high adhesion and deposition capacity onto the crop leaves, which was conducive to the delivery of AZOX into pathogens, and was convenient for application in the agricultural field.

**Figure 9:**
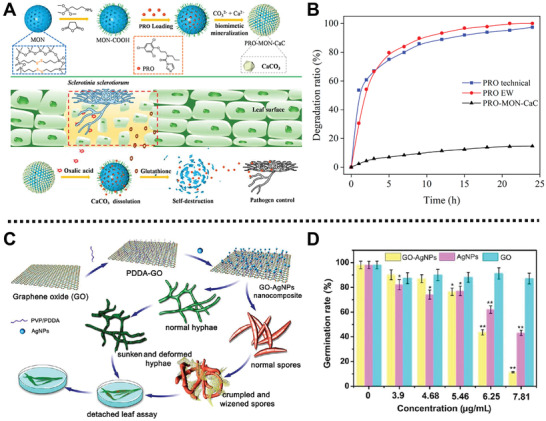
A) Schematic illustration for the preparation process of the bioresponsive PRO‐MON‐CaC platform and its release mechanism in response to *S. sclerotiorum*. B) Ptotostability of PRO‐MON‐CaC, PRO EW, and PRO under UV light. Reproduced with permission.^[^ ^85^ ^]^ Copyright 2020, American Chemical Society. C) Schematic diagram of the fabrication process of GO‐AgNPs composite and its antifungal activity in vitro and in vivo. D) The germination rates of spores treated with GO, AgNPs, and GOAgNPs nanocomposite. Reproduced with permission.^[^ ^86^ ^]^ Copyright 2016, American Chemical Society.

Recently, a microenvironment‐responsive fungicide delivery platform with a UV‐shielding property was designed by Cao and co‐workers for the effective control of *Sclerotinia sclerotiorum* (*S. sclerotiorum*) in plants (**Figure** **9**A).^[^ ^85^ ^]^ Mesoporous organosilica NPs (MONs) were functionalized with carboxyl acid groups, loaded with prochloraz (PRO), and mineralized with calcium carbonate (CaC) to form PRO‐MON‐CaC with significant photostability (Figure 9B). Controlled release experiments showed that PRO‐MON‐CaC exhibited acidic pH‐ and reduction‐responsive capabilities, which was due to the dissolution of the CaC in acidic conditions and the degradation of disulfide bonds under reductive environments, respectively. As an antimicrobial platform, PRO‐MON‐CaC exhibited significant and sustainable fungicidal activity against *S. sclerotiorum*, which could provide a long‐time protection for rapeseed plants. This dual‐responsive fungicide delivery platform with strong fungicidal activity and high photostability opens new prospects for sustainable sclerotinia disease management.

For another instance, Han and co‐workers fabricated a GO‐AgNPs nanocomposite through interfacial electrostatic self‐assembly for the inhibition of phytopathogen, *Fusarium graminearum* (*F. graminearum*) (Figure 9C).^[^ ^86^ ^]^ Negatively charged AgNPs were bound to the surface of positively charged poly(diallyldimethylammonium chloride)‐capped GO (PDDA‐GO) by electrostatic interaction to form GO‐AgNPs. Microdilution method assays showed that GO‐AgNPs displayed brilliant inhibition effects on the germination of *F. graminearum* spores with the minimum inhibitory concentration value of 4.68 µg mL^−1^ (Figure 9D). SEM and TEM images showed that the macroconidia and hyphae were directly contacted and wrapped by GO‐AgNPs nanocomposite, and then became wizened and distorted after incubation for 2 h. Importantly, the excellent fungicidal performance of GO‐AgNPs was mainly attributed to the presence of AgNPs, the release of Ag^+^ and the generation of ROS, which could induce the sporicidal action and inhibit the growth of spores and mycelia. In practical application, when GO‐AgNPs were carried out to administrate wheat seedling infected by *F. graminearum*, only some small‐sized leaf spots were observed, indicating the good protective effect of GO‐AgNPs.

In 2018, Tong et al. developed a NIR‐laser and pH dual‐responsive delivery system (Hy‐GO@PDA) for efficient controlled release of hymexazol (Hy).^[^ ^87^ ^]^ GO support materials were loaded with Hy and then biocompatible and pH‐sensitive PDA coating was wrapped on the surface of GO through oxidative polymerization to form Hy‐GO@PDA. Experimental results showed an enhanced release of Hy (65%) from Hy‐GO@PDA at pH 9.0 within 120 h, which was ascribed to the electrostatic repulsions between the deprotonated amino moieties in PDA and negatively charged hymexazol at alkaline pH. Meanwhile, Hy‐GO@PDA exhibited outstanding hymexazol release upon 808 nm NIR‐laser irradiation. In addition, bioassay and adhesion experimental results showed that Hy‐GO@PDA exhibited good inhibition activity against the growth of *F. oxysporum* and high adhesive attraction to cucumber leaves in simulated rainwater washing conditions, which could effectively improve the utilization and reduce the loss of pesticides.

Interestingly, a sustainable delivery nanoplatform based on layered double hydroxide (LDH) clay nanosheets was reported by Xu and co‐workers for the control of cucumber mosaic virus (CMV) and pepper mild mottle virus (PMMoV).^[^ ^88^ ^]^ Positively charged LDH was loaded with CMV2b‐dsRNA and PMMoVIR54‐dsRNA to form CMV2b‐BioClay and PMMoVIR54‐BioClay with sustained dsRNA release features. The northern blot analysis indicated that CMV2b‐BioClay underwent a slow and sustained release of dsRNA under environmental conditions for 7 d, which was due to the degradation of LDH (caused by the generation of carbonic acid from CO_2_ in the water film on the leaf surface). In addition, confocal microscopy studies showed that Cy3‐labeled CMV2b‐BioClay could be absorbed into the xylem and spongy mesophyll of *A. thaliana* leaves, and CMV2b‐dsRNA–Cy3 could be moved to the non‐treated apical leaves, which indicated that dsRNA could be taken up by the plant cells. Stability test showed that BioClay could withstand rinsing and stay on the leaves, prevent dsRNA from degrading by RNase for 30 d, and protect *Nicotiana tabacum* (*N. tabacum*) plants for 20 d after spraying application. Besides, CMV infected cowpea leaves sprayed with CMV2b‐BioClay showed fewer leaf spot and necrotic regions. PMMoV infected *N. tabacum* leaves treated with PMMoVIR54‐BioClay exhibited outstanding inhibition of PMMoV for 20 d, which was ascribed to the formation of endogenous siRNAs mediated by DICER LIKE‐dependent pathways. This work paves a way to protect crops in actual application by constructing BioClay delivery systems to overcome the drawbacks of genetically modified crops.

## 5 Eco‐Friendly Nanoplatforms for Pest Control

The development of nanotechnology and nanoscience offers flexible strategies to design a new‐generation nanopesticides with controlled release capabilities to improve the effective utilization of pesticides and mitigate the harmful effects on the environment and human health. In recent years, eco‐friendly nanoplatforms equipped with desired features, such as large loading capacity,^[^ ^89^ ^]^ high stability,^[^ ^90^ ^]^ and facile functionalization,^[^ ^91^ ^]^ used for pest control have attracted much interest from biologists and chemists. Moreover, various entity‐functionalized nanoplatforms loaded with insecticides can be sprayed on different parts of the plants or drenched into soil to release active compounds in a slow, sustained, and controlled way under specific stimuli, such as pH, light, temperature, enzyme, and ions, improving the effective utilization of pesticides and reducing the amount of pesticides.^[^ ^36^, ^92^ ^]^. For example, in the presence of *α*‐amylase or cellulase in the salivary glands or guts of insects with chewing mouthparts, the release behavior of the delivery system can be facilitated with dose‐ and concentration‐dependence.^[^ ^22^ ^]^ In this section, we will introduce the applications and advantages of MSNs‐based nanoplatforms for the delivery of insecticide to manage pest (**Table** **4**).

**Table 4:** Eco‐friendly nanoplatforms for crop pest management

Nanoplatform	Cargo	Pest	Stimulus	Ref.
CH–Cu–MCM‐41	CH	–	pH	^[^ ^93^ ^]^
CLAP‐CRFs	CLAP	*P. xylostella* larvae	*α*‐amylase	^[^ ^94^ ^]^
AVM/MSN‐PDA‐M^+^	AVM	Diamondback moths	–	^[^ ^95^ ^]^
AVM loaded HMS@P(GMA‐AA)	AVM	*C. medinalis*	pH	^[^ ^96^ ^]^
THI@HMS@P(NIPAM‐MAA)	THI	*N. lugens*	Temperature	^[^ ^97^ ^]^
Avermectin@MSNs‐ss‐starch NPs	AVM	*P. xylostella* larvae	*α*‐amylase, GSH	^[^ ^98^ ^]^
Me‐Sal‐MCM Me‐Ben‐MCM Me‐Fur‐MCM	Me	*Bactrocera dorsalis*	–	^[^ ^99^ ^]^

For example, a multi‐stimuli‐responsive nanoplatform with sustained release capability based on MSNs was prepared by Chen et al. for the delivery of pesticide.^[^ ^93^ ^]^ Salicylaldehyde‐modified MSNs were loaded with the model drug chlorpyrifos (CH) by the bridge effect of Cu^2+^ to form CH–Cu–MCM‐41. Sustained release tests demonstrated that the release rate of CH from CH–Cu–MCM‐41 was accelerated upon raising temperature, which was ascribed to the weak coordination effect between Schiff base and Cu^2+^ at high temperatures. Moreover, the controlled release of CH from the nanoplatforms also showed sustained release behavior under both acidic and basic conditions.

**Figure 10:**
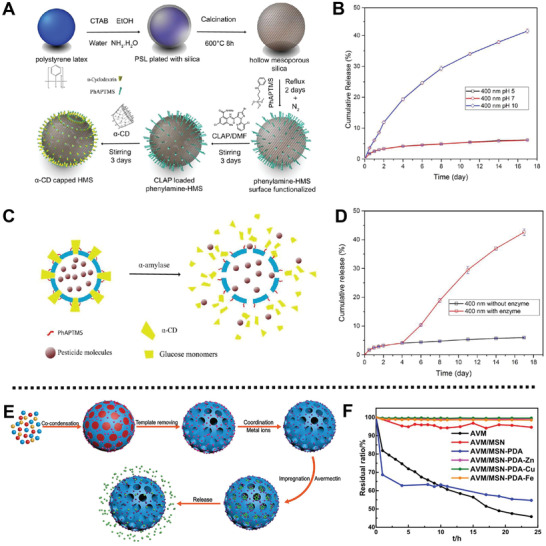
A) Schematic illustration of the fabrication process of CLAP‐CRFs. B) The cumulative release performance of CLAP from CLAP‐CRFs platform at different pH values. C) Schematic description and D) the curve of CLAP release from the CLAP‐CRF triggered by *α*‐amylase. Reproduced with permission.^[^ ^94^ ^]^ Copyright 2017, American Chemical Society. E) Schematic description of the preparation process of AVM/MSN‐PDA‐M^+^. F) Photostability of AVM, AVM/MSN, AVM/MSN‐PDA, and AVM/MSN‐PDA‐M^+^ under UV light for 25 h. Reproduced with permission.^[^ ^95^ ^]^ Copyright 2019, Elsevier.

In addition, Li and co‐workers fabricated controlled release formulations (CRFs) based on hollow MSNs (HMS) carriers and *α*‐cyclodextrin (*α*‐CD) gatekeepers for the delivery of entrapped insecticide in the presence of *α*‐amylase (**Figure** **10**A).^[^ ^94^ ^]^ *N*‐Phenylaminopropyltrimethoxysilane (PhAPTMS) functionalized HMS was loaded with chlorantraniliprole (CLAP) on their surface pore rims, and then capped with *α*‐CD via interactions between *α*‐CD and PhAP group of PhAP‐HMS to obtain CRFs with stimuli‐responsiveness. The release of CLAP from the CLAP‐CRFs showed high cumulative release under the conditions of pH 10 and the presence of *α*‐amylase, which was due to the hydrolysis of Si—O—Si bonds from HMS in alkaline condition and the hydrolyzation of *α*‐CD by *α*‐amylase (Figure 10B–D). Furthermore, the experimental results showed that CLAP‐CRFs could protect CLAP from being decomposed under the UV radiation, which exhibited excellent photostability. Feeding experimental results showed that *α*‐amylase in the saliva of *Plutella xylostella* (*P. xylostella*) could hydrolyze *α*‐CD on the surface of CLAP‐CRFs, thus effectively and continuously releasing CLAP and resulting in long‐term insecticidal activity.

Besides, a photostable and adhesive pesticide delivery system based on MSNs was fabricated by Shen et al. for effective killing diamondback moths.^[^ ^95^ ^]^ After coating with PDA through one‐pot synthesis method, MSN‐PDA was prepared and coordinated with Cu^2+^, Zn^2+^, or Fe^3+^ through the bridge effect, followed by the encapsulation of avermectin (AVM) to form AVM/MSN‐PDA‐M^+^ (AVM/MSN‐PDA‐Cu, AVM/MSN‐PDA‐Zn, and AVM/MSN‐PDA‐Fe) (Figure 10E). Controlled release experiments showed AVM/MSN‐PDA‐Fe and AVM/MSN‐PDA‐Cu with a cumulative release rate less than 15% at pH 4, 7, and 10 within 180 h and AVM/MSN‐PDA‐Zn with a cumulative release rate about 25% under the same conditions, which was ascribed to the increased steric hindrance from the prepared system with metal ion, reducing the release of AVM. Besides, the decomposition rate of AVM from the fabrication of vehicles was decreased, which showed an enhanced photostability under UV light irradiation in comparison with AVM/MSN‐PDA (Figure 10F). Moreover, the insecticide test results demonstrated that the LC50 of AVM/MSN‐PDA‐Cu, AVM/MSN‐PDA‐Fe, and AVM/MSN‐PDA‐Zn were 170.71, 147.60, and 109.79 mg L^−1^, respectively, which was consistent with the controlled release rates of the delivery systems.

**Figure 11:**
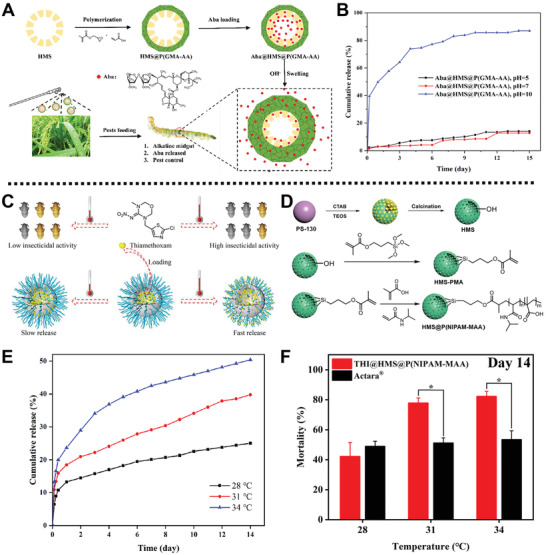
A) Scheme summarization for the preparation process and the insecticidal mechanism of AVM loaded HMS@P(GMA‐AA). B) The cumulative release curve of AVM from the AVM loaded HMS@P(GMA‐AA) platform at different pH values. Reproduced with permission.^[^ ^96^ ^]^ Copyright 2019, Elsevier. C) Schematic diagram of the release mechanism of the thermo‐responsive THI@HMS@P(NIPAM‐MAA) platform. D) The synthesis process of HMS@P(NIPAM‐MAA) system. E) The cumulative release curve of THI from the THI@HMS@P(NIPAM‐MAA) at different temperatures for 14 d. F) The mortality of *N. lugens* treated with THI@HMS@P(GMA‐AA) and Actara for 14 d, respectively. Reproduced with permission.^[^ ^97^ ^]^ Copyright 2020, Elsevier.

Interestingly, a pH‐sensitive nanoplatform based on HMS was fabricated by Li and co‐workers for the effective killing of rice pest *Cnaphalocrocis medinalis* (*C. medinalis*) larvae via long‐term sustainable release of AVM (**Figure** **11**A).^[^ ^96^ ^]^ In this system, poly(glycidyl methacrylate‐co‐acrylic acid) (P(GMA‐AA)) as a pH‐sensitive copolymer gatekeeper was grafted onto the outer layer of the 3‐(trimethoxysilyl)propyl methacrylate (PMA)‐modified HMS by seeded precipitation polymerization to obtain HMS@P(GMA‐AA). Then, AVM was loaded into HMS@P(GMA‐AA) to form AVM loaded HMS@P(GMA‐AA) with a high loading efficiency of 33 wt%. Experimental results demonstrated that the amounts of AVM released from AVM loaded HMS@P(GMA‐AA) were 14%, 13%, and 87% at pH 5, 7, and 10 after 15 d (Figure 11B), which was ascribed to the swelling of the P(GMA‐AA) gatekeeper under alkaline conditions. Furthermore, the decomposition rate of AVM in the as‐prepared nanoplatform was less than 22% within 24 h under UV light irradiation, which showed an enhanced photostability of AVM loaded HMS@P(GMA‐AA). In addition, field trials showed that such a nanoplatform possessed long‐term bioactivity in controlling *C. medinalis* and exhibited little impact on the growth of plants.

Analogously, He and co‐workers prepared a temperature‐controlled insecticide nanoplatform, namely THI@HMS@P(NIPAM‐MAA), for the delivery of thiamethoxam (THI) (Figure 11C).^[^ ^97^ ^]^ PMA with vinyl functional moieties and poly(*N*‐isopropylacrylamide‐co‐methacrylic acid) (P(NIPAM‐MAA)) as a thermos‐responsive copolymer were introduced onto the external surface of HMS‐PMA in turn through seeded precipitation polymerization to obtain HMS@P(NIPAM‐MAA). Subsequently, THI, a positive temperature coefficient insecticide, was loaded into HMS@P(NIPAM‐MAA) via physical mixing to form THI@HMS@P(NIPAM‐MAA) with temperature‐responsive capacity (Figure 11D). The release behavior assays showed that as the temperature increased from 28 °C to 34 °C, P(NIPAM‐MAA) copolymer gradually collapsed, reaching the THI cumulative release of 25.03%, 39.77%, and 50.39%, respectively (Figure 11E). Moreover, in vivo toxicity studies demonstrated that THI@HMS@P(NIPAM‐MAA) with negligible toxicity effects on organisms possessed enhanced insecticidal efficiency to *Nilaparvata lugens* (*N. lugens*) at high temperature (31 and 34 ˚C) (Figure 11F). This work provides a new strategy to develop eco‐friendly nanoplatforms with high photostability, strong adhesion, and enhanced insecticide efficacy for pest control.

Recently, a redox and *α*‐amylase dual stimuli‐responsive insecticide delivery platform based on disulfide‐doped MSNs (MSN‐ss‐OH) was prepared by Cao and co‐workers.^[^ ^98^ ^]^ Amino groups‐functionalized MSN‐ss‐OH were decorated with succinic anhydride, loaded with AVM, and capped with starch‐NH_2_ to form avermectin@MSNs‐ss‐starch NPs. Experimental results showed that 51.3% and 72.2% release of AVM from avermectin@MSNs‐ss‐starch NPs were observed in the presence of GSH and *α*‐amylase after 7 d, which was ascribed to the effectively fracture of disulfide bonds and the degradation of starch on the surface of avermectin@MSNs‐ss‐starch NPs. In addition, three‐instar *P. xylostella* larvae treated with avermectin@MSNs‐ss‐starch NPs (1 mg L^−1^) showed 70% mortality rate after 12 d, showing a high and continuous insecticidal activity.

Interestingly, Chen et al. designed three types of environment‐friendly insecticide delivery systems based on MSNs for the sustained release of methyl eugenol (Me) to lure *Bactrocera dorsalis*.^[^ ^99^ ^]^ Three types of Schiff base (salicylaldimine, furfuralimine, and benzaldehyde imine)‐modified MSNs, denoted as Sal‐MCM, Fur‐MCM, and Ben‐MCM, were used to load Me through electrostatic interaction and *π*–*π* interaction to form Me‐Sal‐MCM, Me‐Ben‐MCM, and Me‐Fur‐MCM, respectively. Accumulated release rates of Me from Me‐Fur‐MCM, Me‐Sal‐MCM, and Me‐Ben‐MCM within 10 h were 56.63% 48.59%, and 37.21%, respectively, which exhibited long‐term sustained release capability of as‐prepared nanoplatforms. In addition, the tests to lure *B. dorsalis* showed that Me‐Fur‐MCM had the highest lure rate of 53.33% (equivalent to 73% of pure Me), which would observably prolong the usage of Me in actual applications.

## 6 Eco‐Friendly Nanoplatforms for Crop Growth Regulation

Long‐term excessive fertilization will leave fertilizer in the soil, which may cause severe soil acidification and environmental degradation.^[^ ^100^ ^]^ It is of a great significance to develop sustainable methods to regulate crops growth when they suffer from environment stress. At present, nanoplatforms as fertilizer delivery systems have become a notable strategy for improving the usage of fertilizers. To achieve efficient utilization of fertilizers, nanoplatforms should satisfy multiple requirements: (i) the nanoplatforms should be biodegradable and eco‐friendly; (ii) the delivery systems should have a high fertilizer loading capacity and flexible response to specific stimuli in the external environment on demand, such as pH, light, temperature, and ions; (iii) the delivery systems should remain stable without external stimuli. In this part, we will highlight the application progress of the nanoplatforms for the regulation of crop growth (**Table** **5**).

**Table 5:** Eco‐friendly nanoplatforms for crop growth regulation

Nanoplatform	Cargo	Stimuli	Plant	Regulation method	Ref.
SA@MSN‐SS‐C10	SA	Redox	*A. thaliana*	Expression of plant pathogen defense gene *PR‐1*	^[^ ^26^ ^]^
Urea‐HA nanohybrids	Urea	–	Rice	Slow release of N	^[^ ^101^ ^]^
SBPCF	Urea fertilizer prills	–	–	Long‐term and slow release of N	^[^ ^102^ ^]^
PHMCN‐Se	Selenate	Anion, pH	Vegetable	Release Se fertilizer	^[^ ^103^ ^]^
GA3‐HMSN/Fe_3_O_4_ NPs	GA3, RhB	pH, BDA, ultrasound	*A. thaliana*, cabbages	Release GA3	^[^ ^16^ ^]^
3D Nanogels	SA	Redox, pH	–	Release SA, and remediate soil	^[^ ^104^ ^]^
NanoU‐NPK	Ca, P, K, NO_3_, urea	–	Durum wheat	Slow release of plant macronutrients	^[^ ^105^ ^]^

In 2015, Kong and co‐workers prepared a GSH‐sensitive nanoplatform based on MSNs for the delivery of phytohormone, SA.^[^ ^26^ ^]^ Thiol groups‐modified MSNs were loaded with SA through the free diffusion and gated with decanethiol as gatekeepers via disulfide bonds to develop SA@MSN‐SS‐C10. In vitro release test, Raman spectra, and thermogravimetric analysis studies showed that the release of SA from SA@MSN‐SS‐C10 was only about 5 µg in the absence of GSH within 24 h. In sharp contrast, an increasing release of SA was observed in the presence of GSH due to the cleavage of disulfide bonds between decanethiol and MSNs. Moreover, in vivo plant test showed that release of the SA from SA@MSN‐SS‐C10 could induce sustained expression of plant pathogen defense gene *PR‐1* in *A. thaliana* for 7 d. Therefore, the smart nanoplatforms with SA sustainable release could prolong the protection time of plants against biotic stress in a controlled manner.

Amaratunga and co‐workers reported a nutrients delivery platform based on a matrix of hydroxyapatite [(Ca_10_(PO_4_)_6_(OH)_2_] NPs (HA NPs) for slow release of nitrogen to crops.^[^ ^101^ ^]^ The urea was coated on the HA NPs by amine‐carbonyl condensation to fabricate urea‐HA nanohybrids. The prepared HA NPs showed brilliant biocompatibility, high loading capacity for urea molecules with 40 wt% nitrogen, ensuring a rich supply of phosphorus and nitrogen source. In the rapid water release test, it was observed that the urea‐HA NPs revealed a slow and sustained release behavior, which released 86% urea within 55 min and demonstrated a long‐term drug release up to 1 week. Subsequently, urea‐HA nanohybrids were developed to study the efficient utilization under actual rice field conditions. The slow release properties of the urea‐HA NPs not only reduced up to 50% in urea consumption, but also improved crops yield (7.9 tons) compared with pure urea group (7.3 tons), which demonstrated that urea‐HA nanohybrids had slow release behavior and remarkable use efficiency of nitrogen (up to 48%).

In addition, a bio‐based polyurethane (BPU) coating containing numerous hydrophilic groups and micro‐holes was reported by Yang and co‐workers to coat fertilizers for the improvement of soil fertility.^[^ ^102^ ^]^ In this case, BPU derived from liquefied wheat straw was used to coat urea prills through a rotating drum approach. Then, the BPU was modified with nano‐silica (NS) and organosilicon (OS) to obtain superhydrophobic BPU coated fertilizer (SBPCF) with increased nanoscale surface roughness and reduced surface energy. Experimental results demonstrated that the amount of nitrogen released from the prepared SBPCF was on the basis of the amount of OS in the SBPCF, when 10%, 20%, and 30% OS was in the SBPCF, it took 28, 28, and 21 d to release 80% nitrogen, respectively. Such a coating strategy provides a way to prepare nanoplatforms with controlled release of nutrients.

**Figure 12:**
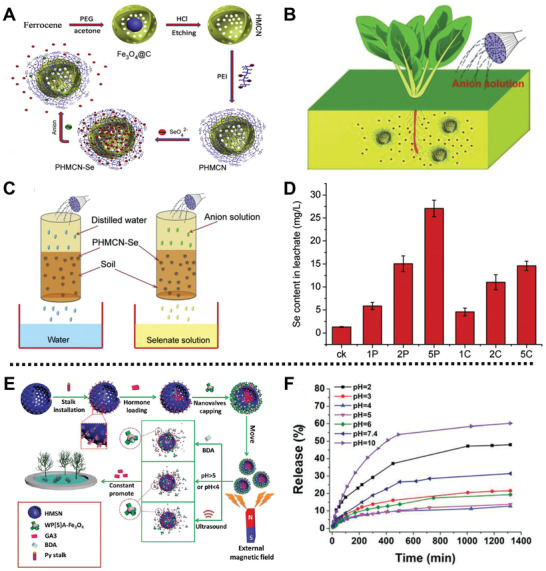
A) Scheme summarization of the synthesis process of anion‐responsive Se fertilizer CRS. B) The release behavior of the Se fertilizer CRS in response to anion in the soil. C) Schematic illustration of the release behavior of PHMCN‐Se in response to anion in leaching experiment. D) The amount of Se release from the Se fertilizer CRS in the soil under different concentration and valence of anion. Reproduced with permission.^[^ ^103^ ^]^ Copyright 2018, Elsevier. E) Schematic description of the fabrication process of the GA3‐HMSN/Fe_3_O_4_ platform and its application in response to multi‐stimuli for the regulation of plant growth. F) The cumulative release curve of RhB from RhB‐HMSN/Fe_3_O_4_ at different pH values. Reproduced with permission.^[^ ^16^ ^]^ Copyright 2019, Royal Society of Chemistry.

In recent years, carbon‐based fertilizer delivery systems with the advantage of sustainable release have drawn an increasing attention. For example, anion‐responsive hollow mesoporous carbon NPs (HMCNs) were prepared by Wu and co‐workers, for the delivery of selenium fertilizer.^[^ ^103^ ^]^ In this case, HMCNs prepared by the decomposition of ferrocene were etched with acid solution and modified with cationic polymer (polyethyleneimine) as gatekeepers to obtain the PHMCN. Then, selenium fertilizer was loaded in the PHMCN carriers through electrostatic interaction between polyethyleneimine chain and selenium to form PHMCN‐Se delivery platform with a loading capacity of 389 mg g^−1^ (**Figure** **12**A). The release behavior studies showed that the release of selenate from PHMCN‐Se at neutral pH was negligible, whereas a marked release was observed under alkaline condition, which was ascribed to deprotonation of amino group detached selenate from polyethyleneimine molecules. Meanwhile, the release of selenate from PHMCN‐Se could also be stimulated by anions including PO_4_^3+^, CO_3_^2−^, and OH^−^, and the release amount of selenate was related to the valence and concentration of anions (Figure 12B–D). Botany assays results indicated that the yield of vegetables could be increased by the combination of PHMCN‐Se and phosphate, producing the highest yields of vegetables at the optimum concentration of 5 mmol L^−1^ phosphate.

Interestingly, a triple‐stimuli responsive gibberellin acid (GA3) delivery system based on HMS and water soluble carboxylatopillar[5]arene ammonium (WP[5]A)‐functionalized Fe_3_O_4_ NPs (WP[5]A‐Fe_3_O_4_) was prepared by us to promote the growth of *A. thaliana* and cabbages (Figure 12E).^[^ ^16^ ^]^ In this case, pyridine‐modified HMS was loaded with GA3/rhodamine B (RhB) and capped with WP[5]A‐Fe_3_O_4_ nanovalves through host‐guest interactions to form GA3‐HMSN/Fe_3_O_4_ delivery platform. This system exhibited a bidirectional pH‐responsive capability under pH<4 or pH > 5 conditions (Figure 12F), and other stimuli‐responsive capabilities including 1,4‐butanediamine (BDA) and ultrasound. Germination assay results showed that the lengths of stems and roots of cabbages increased obviously after GA3‐HMSN/Fe_3_O_4_ treatment for 5 d. Similarly, the lengths of stems and roots of *A. thaliana* treated with GA3‐HMSN/Fe_3_O_4_ also increased significantly after 16 d of culture, which indicated that the GA3‐HMSN/Fe_3_O_4_ system could release GA3 in a controlled manner to promote the growth of plants.

Subsequently, Xiao and co‐workers designed a pH‐ and redox‐responsive phytohormone delivery system based on cellulose nanogel, which was constructed from hydrophobic carboxymethyl cellulose (HCMC) and 3,3′‐dithiobis(propionohydrazide) for simultaneous release of SA and remediation of soil.^[^ ^104^ ^]^ In this system, palmitic chloride was grafted on the carboxymethyl cellulose to obtain the HCMC through esterification, and then acylhydrazone bond was introduced in the structure of HCMC to form pH‐ and redox‐responsive 3D nanogel. The prepared 3D network of nanogels delivery system could dissolve in acidic conditions and/or in the presence of redox reagent, for the destruction of acylhydrazone and disulfide bonds from the nanogels contributed to the pH‐responsiveness and redox‐responsiveness. Under neutral conditions, 23% SA was registered, whereas 80% SA was observed at pH 3.5 within 4 h, which was due to the destruction of acylhydrazone in acidic conditions. Moreover, in the presence of GSH, hydrolysis of disulfide bonds could also lead to the controlled release of SA from the nanogels. Furthermore, in the simulated experiment of soil leachate, the presence of carboxyl and thiol groups in the nanogels effectively induced the complexation of Cu^2+^ in the soil, which would be efficient removal 89% of Cu^2+^ to remediate agricultural soil.

As a major source of nutrients in crops, potassium (K), nitrogen (N), and phosphorus (P) play key roles in providing macronutrients for crop growth. Therefore, regulating the supply of NPK to crops is an effective strategy to reduce the use of fertilizers and stabilize the yield of crops. A nanofertilizer (nanoU‐NPK) doped with K, N, urea, and calcium phosphate was reported by Delgado‐López and co‐workers for the supply of macronutrients to durum wheat via a controlled manner and management the growth of the crop.^[^ ^105^ ^]^ The experimental results showed the release of Ca, P, K, urea, and NO_3_^−^ from the nanoU‐NPK in water demonstrated a slow release behavior of 27, 6.2, 41, 315, and 134 mg L^−1^, respectively, after 1 week. Besides, in the leaching experiment, 90% of crystalline urea pellet was leached within 1 h in a vertical column simulated solid medium. As a clear contrast, 83% urea from the nanoU‐NPK was leached away after 6 h, for the structure of nanoU‐NPK slowed down the release of urea. Besides, the durum wheat treated with the prepared nanoU‐NPK (N, 75 kg ha^−1^) in growth chambers demonstrated that the amount of N was greatly reduced by 40% than conventional fertilizers diammonium hydrogen phosphate (N, 150 kg ha^−1^), whereas similar kernel weights were obtained. This work provides a possibility to construct controlled‐release multi‐nutrient nanosystems with biodegradability and biocompatibility for efficient and sustainable delivery macronutrients of crops.

## 7 Conclusion and Perspective

We have overviewed the recent advanced developments and achievements of eco‐friendly platforms for sensitive and rapid on‐site detection of agricultural chemicals and mycotoxins, control of plant pathogens, on‐demand delivery of agricultural chemicals to manage pest, and regulation of crops growth in a sustained manner. So far, researchers have been dedicated to developing efficient, stable, multifunctional, and environment‐friendly solutions to achieve food security together with “Zero hunger.” Therefore, numerous advanced nanoplatforms with outstanding properties have been fabricated to fuel new applications.

As abovementioned, metal/metal oxide NPs as functional materials combined with SERS technique, colorimetric and spectroscopic strategies, thermal calcination method, LFI, ICTS and others, can realize on‐site detection of agricultural chemicals and mycotoxins in a rapid, effective, and convenient way.

Besides, MSNs‐based materials with multiple features, including high loading capacity, good biocompatibility, and easy functionalization, have attract extensive attention from scientists to be used as carriers to construct pesticides/fertilizers delivery systems. Meanwhile, MSNs functionalized with polymers such as P(NIPAM‐MAA),^[^ ^97^ ^]^ P(GMA‐AA),^[^ ^96^ ^]^ PDA,^[^ ^84^, ^87^, ^95^ ^]^ possess high photostability against ultraviolet light, and strong adhesivity onto the crop leaves against rainwater erosion.^[^ ^98^ ^]^ Besides, various stimuli, such as pH, enzyme, temperature, redox reactions, and ions, have been used as external triggers to open the nanovalves gated on the surface of MSNs for achieving controlled release of pesticides, enhancing the utilization efficiency of pesticides/fertilizers and providing precision farming methods. These superiorities of MSNs are beneficial to enhancing the long‐term bioactivity of pesticides in nanocarriers, providing a new candidate for green agriculture.

Although eco‐friendly nanomaterials exhibit excellent performance for controlling pathogenic microorganism, detecting or releasing agricultural chemicals, they still face severe challenges of potential safety concerns on agricultural products and long‐term effects on the environment.^[^ ^106^ ^]^ First, residual nanomaterials are one of the most crucial problems in the crop products exposed to the engineered NPs. When people consume food treated with NPs, nanomaterials are accumulated in the leaves, roots, and other parts of the plants, resulting in a potential problem to the human health.^[^ ^107^ ^]^ Therefore, the toxicity of various nanomaterials requires a comprehensive assessment in vivo, including acute toxicity, long‐term toxicity, immunotoxicity, and genotoxicity. Second, more studies should be carried out for long‐term monitoring the degradation process of nanomaterials and the ultimate impact on environment.^[^ ^108^ ^]^ Furthermore, improving the stability of nanoplatforms to reduce premature release of cargos during storage period is another key challenge. Therefore, effective gatekeepers should be designed and introduced onto the surface of nanoplatforms to lock cargos in the inner porous of the support under normal environment to realize long‐term protection of pesticides/fertilizers.^[^ ^16^, ^94^ ^]^ Although eco‐friendly nanomaterials still face many challenges in actual agricultural applications, various nanoplatforms with advanced properties have been developed to meet a series of requirements in the process of green agricultural revolution, which provide security guarantees and powerful engines for the sustainable development of contemporary agriculture.

## Conflict of Interest

The authors declare no conflict of interest.
